# Supplementary material for: MultiGreen: A multiplexing architecture for GreenGate cloning
Source: PLoS One. 2024 Sep 18;19(9):e0306008. doi: 10.1371/journal.pone.0306008 (PMC11410190; doi:10.1371/journal.pone.0306008)
Supplement: S1 Method — (PDF) [file pone.0306008.s001.pdf]

MultiGreen: Supplementary Methods

Level 1 to level 0 module reversion

Level 1 MultiGreen 2.0 assemblies can optionally be reverted to GreenGate level 0 modules via conventional T4 restriction ligation, as level 1 MultiGreen 2.0 assemblies contain conventional GreenGate overhangs. To revert level 1 MultiGreen 2.0 assemblies back to level 0 modules, 1 µg of level 1 assemblies can be digested with 1 µL of *Esp*3I, 1 µL of rCutSmart buffer, in a 10 µL reaction for 3h at 37°C to ensure complete digestion. Following digestion, the reaction mix should be heat-inactivated at 80°C for 20 minutes to deactivate remaining *Esp*3I. One µL of heat-inactivated digest can then be combined in a 5-µL ligation with 1 µL of *Bsa*I-HFv2 pre-digested compatible entry vector normalized to 50 ng·µL^{-1}, 0.5 µL of T4 DNA ligase, and 1 µL of 5x rapid ligation buffer (ThermoFisher Scientific, Waltham, MA, #K1422). The ligation is incubated on the benchtop for 5-10 minutes, and directly transformed into *E. coli*, followed by selection on LB ampicillin plates for correctly reverted modules.

GFP bacterial promoter validation

GFP reporter constructs of varying promoters were generated through conventional GreenGate assembly of level 0 parts listed in S2 Table. Four different constructs, pVP272, pVP284, pVP285, and pVP286, were assembled using the four different promoters, pGG-A-PJ23119:PGLPT-C/pVP217 derived from pEN-L1-PJ23119-Bsa1-PglpT-sfGFP-TrrfB-Bsa1-Scaf-L2 [1], pGG-A-PJ23100:B0030-C/pVP213 derived from the PJ23100 Anderson promoter [2] and B0030 RBS[3], pGG-A-PJ23119:PGLPT-ATG-C/pVP275, and pGG-A-PJ23100:B0030-ATG-C/pVP276, respectively to drive expression of eGFP [4]. The AC promoter modules, CD eGFP, pGG-D-rrnbT1-F/pVP223 terminator, and pGG-F-dummy-G linker module were

assembled into the spectinomycin-resistant pVP083 MultiGreen intermediary vector using the NEBridge ligase master mix. The plasmids were transformed into DH5α, sequenced, and stored as glycerol stocks at -80°C prior to evaluation.

One day prior to evaluation, 3 mL liquid LB cultures were inoculated in triplicate from each glycerol stock and a DH5α control for 16-18 h incubation and shaking at 37°C. After incubation, 1 mL of culture was centrifuged at 8000 x *g* for 1.5 min. The supernatant was decanted, and the pellet resuspended in 1 mL of PBS (137 mM NaCl, 2.7 mM KCl, 10 mM Na_2HPO_4, 1.8 mM KH_2PO_4). The PBS washing was repeated 1x. After the second wash, pellets were resuspended to a final OD600 of 1.0, and 200 μL of each culture were loaded into an untreated black flat bottom 96-well microplate (Caplugs Evergreen, Buffalo, NY, #290-8195-Z1F). GFP fluorescence was collected on a Biotek Synergy2 plate reader through a 485/20 nm excitation filter, 510 nm dichroic mirror, and 516/20nm emission filter. Emission data for two, triplicated biological replicates performed on separate days were imported to GraphPad Prism 10 for macOS version 10.2.0 for plotting and analysis (S1 Fig).

References

1. Rodrigues, S.D., et al., *Efficient CRISPR-mediated base editing in Agrobacterium spp.* Proceedings of the National Academy of Sciences, 2021. **118**(2): p. e2013338118.

2. Anderson, J. *Part:BBa_J23100*. 2006; Available from: https://parts.igem.org/Part:BBa_J23100.

3. Vinay S Mahajan, V.D.M., Brian Chow, Alexander D Wissner-Gross and Peter Carr *Part:BBa_B0030*. 2003 [cited 2024; Available from: https://parts.igem.org/Part:BBa_B0030.

4. Cormack, B.P., R.H. Valdivia, and S. Falkow, *FACS-optimized mutants of the green fluorescent protein (GFP).* Gene, 1996. **173**(1): p. 33-38.
