# Supplementary figures and images for: MultiGreen: A multiplexing architecture for GreenGate cloning
Source: PLoS One. 2024 Sep 18;19(9):e0306008. doi: 10.1371/journal.pone.0306008 (PMC11410190; doi:10.1371/journal.pone.0306008)

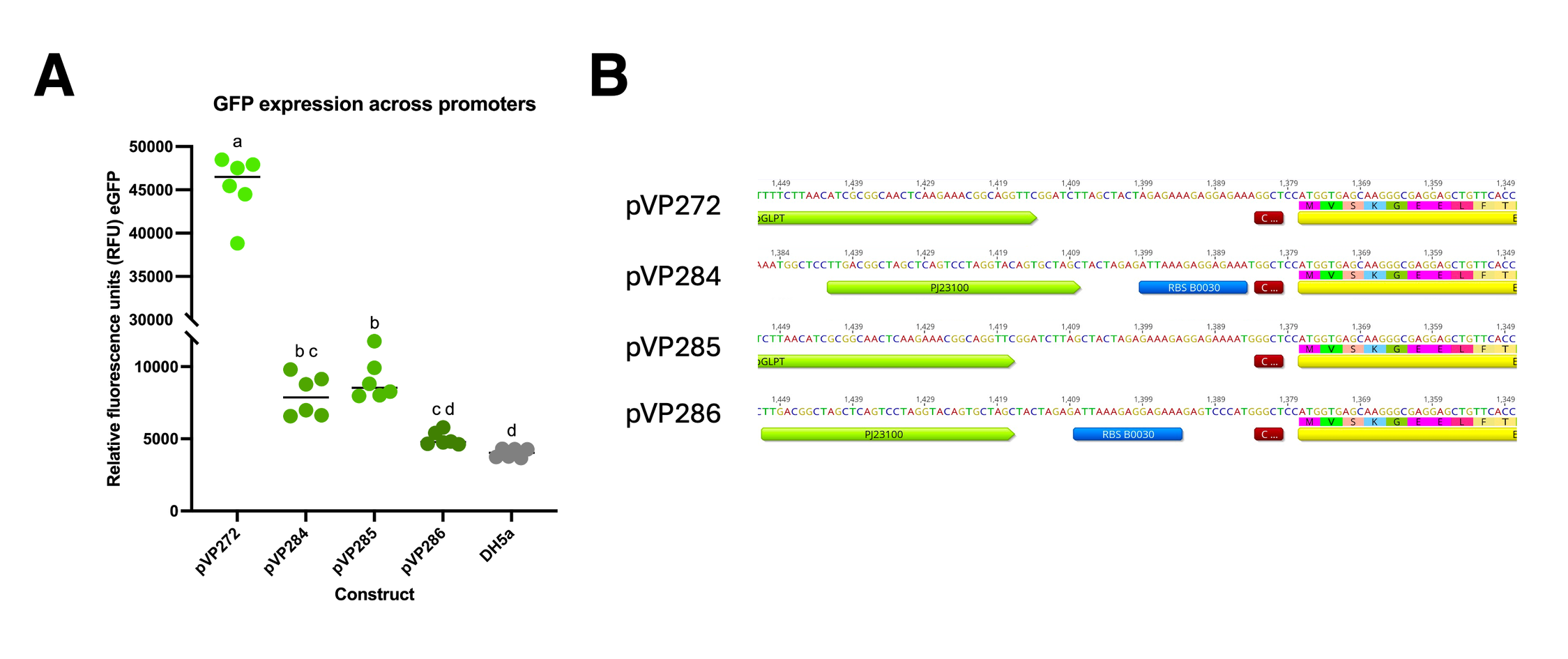

Supplement: S1 Fig — A) Quantitative measurement of GFP on a Synergy2 microplate reader collected with a 485/20 nm excitation filter, 510 nm dichroic mirror, and a 516/20nm emission filter. B) Alignment of the four constructs. pVP272 contains the progenitor promoter module of pVP285; pVP284 contains the progenitor promoter module of pVP286. Level 0 promoter modules used in pVP284 and pVP286 remove out of frame ATG codons after the RBS, introducing Met-gly-ser residues within the A-C level 0 module itself. (TIF) [file pone.0306008.s003.tif]
